# Parent-embryo acoustic communication: a specialised heat vocalisation allowing embryonic eavesdropping

**DOI:** 10.1038/s41598-018-35853-y

**Published:** 2018-12-07

**Authors:** Mylene M. Mariette, Anaïs Pessato, William A. Buttemer, Andrew E. McKechnie, Eve Udino, Rodney N. Collins, Alizée Meillère, Andrew T. D. Bennett, Katherine L. Buchanan

**Affiliations:** 1 Centre for Integrative Ecology, School of Life & Environmental Sciences, Deakin University Geelong, Geelong, Australia; 2 School of Biological Sciences, University of Wollongong, Wollongong, Australia; 3 DST-NRF Centre of Excellence at the FitzPatrick Institute, Department of Zoology and Entomology, University of Pretoria, Pretoria, South Africa; 4 South African Research Chair in Conservation Physiology, National Zoological Garden, South African National Biodiversity Institute, P.O. Box 754, Pretoria, 0001 South Africa

## Abstract

Sound is arguably the external cue most accessible to embryos of many species, and as such may constitute an unrivalled source of early information. Recent evidence shows that prenatal sounds, similarly to maternal effects, may shape developmental trajectories. Establishing whether parental vocalisations are signals directed at embryos, or parental cues on which embryos eavesdrop, can elucidate whether parents or embryos control developmental outcomes. Prenatal exposure to a characteristic heat-related parental call was recently shown to alter zebra finch growth and fitness. Here, we test the ecological context of this behaviour in the wild, and assess the information value and specificity of this vocalisation for an embryonic audience. We show that wild zebra finches also produce this characteristic call, only at high temperatures. In addition, in the lab, we demonstrate experimentally that calling is specifically triggered by high air temperatures, can occur without an embryonic audience, and importantly, is predicted by individuals’ body mass. Overall, our findings reveal a specialised heat vocalisation that enables embryonic eavesdropping, by indicating high ambient temperatures, and parents’ capacity to cope with such conditions. This challenges the traditional view of embryos as passive agents of their development, and opens exciting research avenues on avian adaptation to extreme heat.

## Introduction

Prenatal acoustic communication occurs in a wide variety of taxa, ranging from crocodiles to humans^1–3^. Among birds, prenatal acoustic communication has long been known to fulfil several functions, from imprinting and individual recognition, to synchronisation of hatching^4–6^. In particular, in precocial species, embryos respond to parental vocalisations by producing clicking sound or vocalisations^2^ and even learn to discriminate individual maternal calls encountered prenatally from those of unfamiliar females^7–10^. More recently in altricial songbirds, parental vocalisations during incubation have been shown to vary according to the biotic and abiotic context such as parasitic cuckoo presence^11^ or ambient temperature^12^, and may accordingly play a role in brood parasitism avoidance^13,14^ or heat adaptation^12^. For example, in two species of fairy wrens, nestlings have been found to produce begging calls resembling part of the maternal incubation call, which is thought to allow provisioning parents to discriminate against dissimilarly-sounding cuckoo nestlings^13–15^. Surprisingly however, in spite of this diversity of functions across species, whether parents produce calls specifically to signal to embryos has rarely been questioned. Yet, similarly to other communication systems, establishing the circumstances of signal production is essential for understanding the fitness benefits that signals may confer on senders and receivers^16^, and therefore for elucidating the evolution of prenatal acoustic communication.

Prenatal acoustic stimuli alone were recently found to also alter post-hatch developmental trajectories and long-term fitness, thus revealing prenatal communication as a novel mechanism for developmental programming^12^. Such a mechanism offers a unique opportunity to address the long-standing question of the extent of parental control over embryonic environment, and therefore of the evolutionary consequences of maternal effects^17,18^. Indeed, with few exceptions^19,20^, the adaptive value of maternal effects and whether they hinder or facilitate adaptation to rapidly changing environments, remain unclear^17,18,21^. This partly reflects the difficulty of establishing whether mothers can actively control hormone and nutrient levels reaching their embryos, in relation to environmental conditions^22,23^. By contrast, the utterance of parental vocalisations, and their specific environmental triggers and functions, can be readily established experimentally.

Recently, wild-derived zebra finch parents were shown to produce a characteristic call while incubating eggs at high air temperatures in outdoor aviaries^12^, and prenatal experimental exposure to this specific vocalisation to adaptively affect subsequent nestling growth and begging behaviour in a temperature-dependent manner^12^. These findings suggest that prenatal acoustic communication may provide a mechanism for birds to prepare their offspring for high ambient temperatures, at least in an arid-adapted species. Understanding how this putative signal may have evolved requires assessing the ecological context of this behaviour in wild populations under natural climatic conditions, and establishing whether calling is exclusively directed to an embryonic audience and reliably indicates hot conditions.

## Results and Discussion

### Fluctuating high temperatures trigger in-nest calling in wild zebra finches

**Figure 1:**
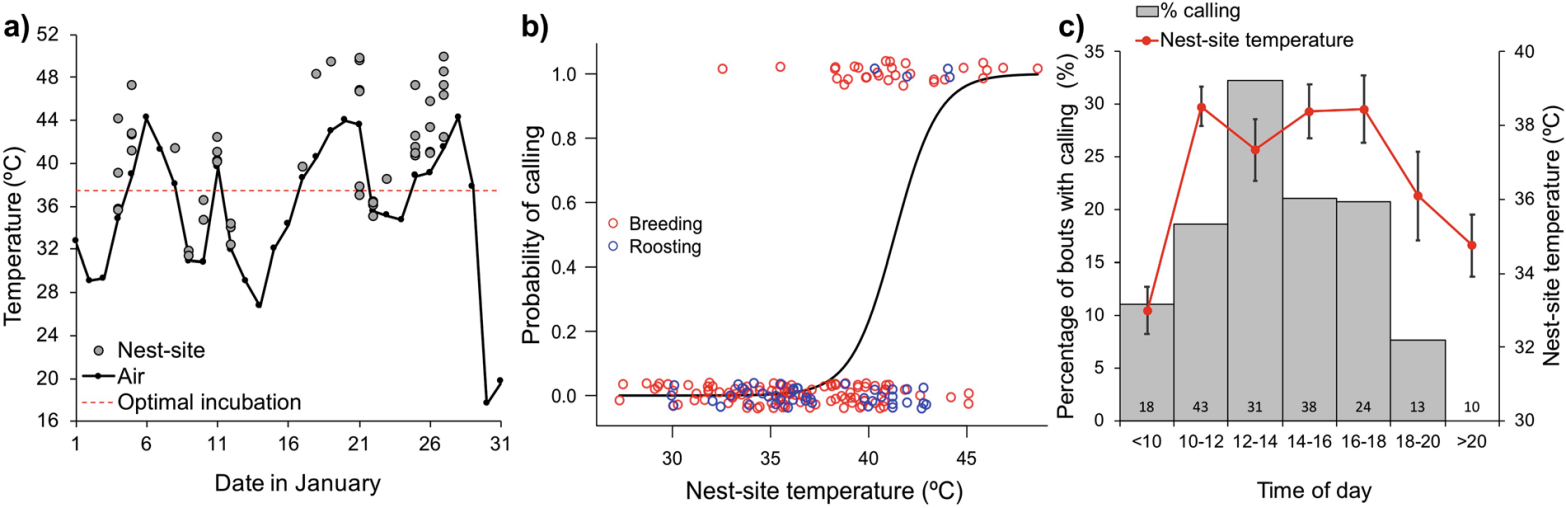
(**a**) Maximum daily air temperature (black line) and maximum nest-site temperature during recording (grey circles), fluctuating above and below the optimal incubation temperature (red dotted line), in January 2018 (summer) at Bimbowrie Conservation Park, South Australia. (**b**) Calling probability per bout in wild zebra finches in breeding and roost nests in relation to nest-site temperature (n = 177 in-nest bouts, for 40 individuals, in 21 nests). (**c**) Percentage of bouts where calling occurred throughout the day in two-hour intervals and corresponding average (±s.e.) nest-site temperature during recording. Numbers at the base of each bar indicate sample sizes (*n* of bouts) per time interval. All bouts occurred in daylight except for those in the >20 bar, which occurred after dusk.

We recorded vocalisations of wild zebra finches in active natural nests (*n* = 177 in-nest bouts (i.e. uninterrupted presence of the individual in the nest), from 40 individuals in 21 nests) in the Australian semi-arid zone during summer (January 2018). Ambient temperatures (mean daily maximum = 36.8 °C; Fig. 1a) fluctuated widely around the optimal incubation temperature of 37.5 °C, both within and between days, varying from night-time minima of 12.8 to 29.2 °C (mean 21.2 °C) to daytime maxima of 20.4 to 44.4 °C. Nest-site temperatures (i.e. air temperature surrounding the nest) tracked ambient temperatures, but were up to 10 °C higher on account of solar radiation, reaching a maximum of 49.9 °C (Fig. 1a). As with their captive counterparts, we found that wild zebra finches under natural field conditions produced a characteristic call at high nest-site temperatures above 35 °C (*Est* = 3.84, *s.e*. = 1.07, *z* = 3.59, *P* = 0.0003; Fig. 1b). Nest-site temperature better predicted calling occurrence than did daily maximum air temperature (ΔAIC = 15.9). There was no difference between males and females in the probability of calling (*Est* = 0.57, *s.e*. = 1.02, *z* = 0.56, *P* = 0.57) or in the temperature triggering calling (interaction sex × nest-site temperature: *Est* = 2.11, *s.e*. = 2.00, *z* = 1.06, *P* = 0.29). Also, after controlling for nest-site temperature, calling probability decreased throughout the day (linear: *Est* = −1.74, *s.e*. = 0.67, *z* = −2.61, *P* = 0.009; quadratic: *Est* = 1.08, *s.e*. = 0.50, *z* = 2.15, *P* = 0.032; Fig. 1c). Overall, our results show that parental vocalisations can indicate short-term variations in temperature, and potentially heat waves, to embryos.

### Calling can occur without an embryonic audience, in wild and captive zebra finches

In both zebra finches and another songbird species, parents were found to produce particular in-nest vocalisations only during the last third of the incubation period, but not on earlier-stage eggs^12,13^. This may be because embryos can perceive these calls only at an advanced stage of development, or because of other unknown constraints on the parents or the embryos at different stages of incubation. Therefore, to unequivocally establish whether parents specifically emit these calls for embryos, or whether embryos eavesdrop on parental behaviour, it is necessary to test whether parents only call when an embryonic audience is present. To do this in wild birds, we took advantage of zebra finches’ use of roost nests, including during the day, to record the vocalisations of non-breeding adults under the same environmental conditions as adults incubating eggs in breeding nests. We found that non-incubating adults in roost nests also called at high nest-site temperatures, although less often than incubating adults (*Est* = −3.94, *s.e*. = 1.57, *z* = −2.51, *P* = 0.012; Fig. 1b). This suggests that heat-triggered vocalizations are not solely directed at embryos, nor only given by breeding adults.

**Figure 2:**
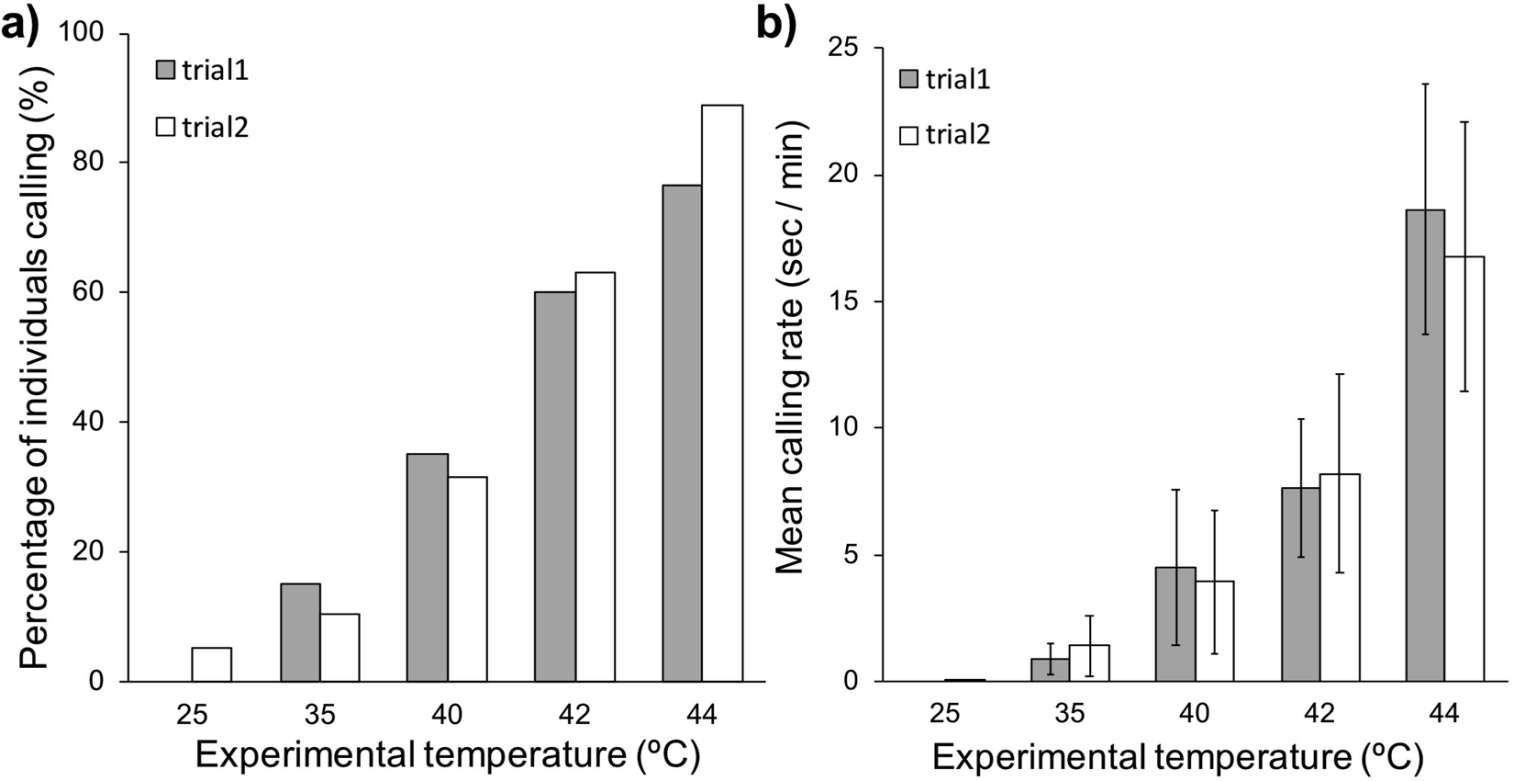
(**a**) Percentage of individuals calling and (**b**), mean (±s.e.) calling rate (i.e. time (in seconds) spent calling per minute) at increasing air temperatures in experimental chamber, for trials one and two per individual, in wild-derived zebra finches (*n* = 20 trials 1, *n* = 19 trials 2, with 20 individuals).

Therefore, to determine experimentally whether non-breeding adults call in a temperature-dependent manner, we recorded vocalisations of wild-derived adults in the laboratory, when placed individually in a small (1.5 L) heated chamber (*n* = 39 recordings on 20 individuals) to simulate an acute heat exposure. After acclimation at 25 °C for 25 or 45 minutes, the air temperature in the chamber was increased over a two-hour period in a stepwise manner, to 35 °C for 30 minutes and then in 2 °C increments for 20-minute stages from 40 to 44 °C. Again, we found that individuals called in the absence of eggs or even a nest (Fig. 2), confirming experimentally that neither the presence of developed eggs or breeding status are necessary for calling to occur. In addition, regardless of the length of their exposure to 25 °C before increasing the temperature, adults called only at air temperatures above 35 °C (except for one bird calling briefly at 25 °C; *Est* = 10.2, *s.e*. = 3.09, *z* = 3.32, *P* = 0.0009; Fig. 2a,b). These results suggest that high air temperature – or the associated heat-stress response – triggers calling in zebra finches, while other potential stressors such as handling and isolation (most prevalent at the start of the test at 25 °C) do not. Moreover, similarly to wild birds, after controlling for temperature, individuals under experimental conditions were more likely to call in the morning than the afternoon, although only marginally so (*Est* = −1.39, *s.e*. = 0.75, *z* = −1.84, *P* = 0.065). Furthermore, like calling occurrence, calling rate (i.e. time spent calling per minute) also increased with air temperature (*Est* = 2.28, *s.e*. = 0.10, *z* = 22.3, *P* < 0.0001; Fig. 2b) and decreased in the afternoon (*Est* = −0.48, *s.e*. = 0.10, *z* = −4.78, *P* < 0.0001).

### Calling as an individual-specific indicator of heat stress

**Figure 3:**
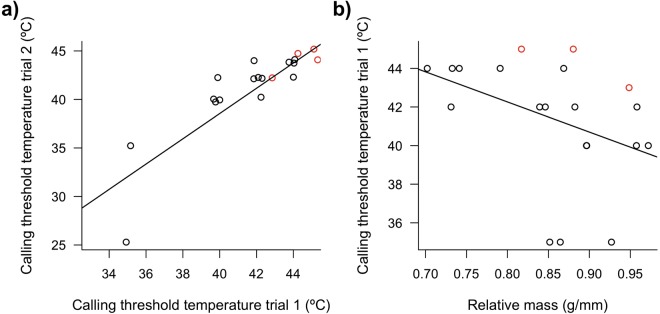
(**a**) Within individual correlation in the air temperature threshold (i.e. lowest temperature) triggering calling in their first and second trials. Points are slightly jittered for clarity. The points in red are for individuals that did not call during one (n = 3 individuals) or both (n = 1 individual) trials; we assumed these non-calling individuals had not reached their calling threshold, and used 1 °C above the maximal air temperature tested as their hypothetical calling threshold. Thresholds were highly correlated between trials within individuals, with or without these 4 extrapolated individuals (ρ = 0.87, *P* < 0.0001, *n* = 19 and ρ = 0.84, *P* < 0.0001, *n* = 15, respectively). (**b**) Air temperature threshold for calling in relation to individual relative body mass (i.e. mass in g divided by tarsus length in mm). As in (**a**) points in red are for individuals that did not call (in trial 1). Calling threshold was related to relative mass with or without these individuals (LM: *t* = −2.66, *P* = 0.017, *n* = 20; and *t* = −2.68, *P* = 0.019, *n* = 17; respectively) and time of day had a marginal effect (with all individuals only: *t* = 1.90, *P* = 0.076).

Despite the clear effect of temperature, and while the wild and captive populations did not dramatically differ, there was noticeable inter-individual variation in the temperature threshold triggering calling (Figs 1b, 2a, 3a). However, when the same individuals were tested twice, one to three weeks apart, the temperature threshold triggering calling in the chamber was highly repeatable within individuals (ρ = 0.87, *P* < 0.0001; Fig. 3a). Importantly, as predicted by Bergmann’s rule and the expectation that smaller body size facilitates heat loss^24,25^, the temperature calling threshold was predicted by body mass, with heavier individuals (relative to skeletal size) commencing calling at lower air temperature (*LM*: *t* = −2.66, *P* = 0.017, Fig. 3b). Our results therefore raise the possibility that calling may be an honest indicator of heat-stress in zebra finches, and provides information to embryos, not only about current high temperature extremes, but also about the capacity of their parents to cope with such conditions. Such individual-specific information may be particularly relevant for embryos, for predicting post-hatch conditions in the nest and/or parental provisioning during hot conditions.

## Conclusion

Given the effects of parental calling demonstrated previously on nestling development and fitness in zebra finches^12^, our findings reported here indicate that embryos can gain information about the external environment by eavesdropping on parental heat-triggered vocalisations, and then modulate their development accordingly. This contrasts with the traditional paradigm considering embryos as passive players under the full control of prenatal maternal effects, and instead points to a more active role of embryos in determining their own developmental trajectories^26^. Our findings therefore align with emerging evidence suggesting that avian embryonic hormone metabolism may interact with the endocrine environment provided by the mother to alter development^27–29^. Furthermore, our results highlight the importance of the acoustic channel in enabling embryos to collect direct information about the external environment, thereby empowering them in the potential parent-offspring conflict occurring prenatally.

Our results also raise interesting questions about the possible adaptive functions of this vocalisation for the adults, particularly given its association with body mass. Stress is known to alter the frequency (i.e. pitch) of common vocalisations such as contact or begging calls^30,31^. However, our study is to our knowledge, the first evidence that adult birds have a specialised vocalisation type triggered by heat. It is possible that this vocalisation has a communicative function between partners or colony members, or it might be associated with an unknown thermoregulatory behaviour. Overall, our findings have implications for understanding the evolution of maternal effects and prenatal acoustic communication, as well as for uncovering avian strategies to cope with extreme heat in arid-adapted species.

## Methods

### Study species and study site

The zebra finch distribution encompasses most of the Australian continent and it is particularly common in the arid and semi-arid zone^32^. It is nomadic, mostly colonial, and breeds opportunistically throughout the year^33,34^. Zebra finches have life-long social monogamy and highly coordinated parental care, with both parents sharing incubation duties^32,35^.

The field study was conducted at Bimbowrie Conservation Park, South Australia (GPS: −32.048935; 140.161977), located in the arid interior. The study area consisted of open shrubland on a flood plain dominated by Acacia species (*Acacia victoriae, A. aneura, A. oswaldii and A. carneorum*) and inland rosewood trees (*Alectryion oleifolius*) typically hosting thick mistletoe clumps (*Lysiana exocarpi*). The study area extended between two artificial watering points used by zebra finches, 7 km apart. Rain at the field site is scarce and highly unpredictable (average annual rainfall 260 mm), whereas temperature varies seasonally. Sun rose at 6:15am and set at 8:10 pm in January.

### Nest audio recording in the wild

In January 2018, all trees and bushes suitable for zebra finch nesting in the study area were searched for the presence of breeding or roost nests. Zebra finch nests are made of fine twigs and grasses, and comprises of an enclosed chamber accessed via an entrance tunnel^32^. Roost nests (unlike roosting platforms) are identical to breeding nests, except that the entrance is typically larger^32^. The presence of eggs in the nest was established using a small telescoping mirror. However, embryonic age at recording could not be assessed, because eggs could not be taken out for candelling, all nests but one were found after egg laying was complete, and only 18% of nests successfully hatched.

For audio recording, a tie-clip microphone (Sennheiser MKE 2 P Germany) was put at the top of the nesting chamber, just above the incubating or roosting bird (except for 2 nests where the roof was not accessible). The microphone was connected to a Zoom recorder (H4nSP or H6) hidden in the vegetation at least 2 m from the nest. Two thermo-hygrometer data-loggers (Minnow 1.0, Senonics, USA) were hung in the branches, within 30 cm of the nest, positioned to receive comparable solar radiation to the nest itself. We did not place them inside the chamber, to avoid the bird position affecting temperature measurements. “Nest-site temperature” was the average of the temperature recorded by the two data-loggers. Relative humidity at the nest-site remained low throughout the study period and vapour pressure deficit (i.e, the gradient in vapour density between the birds and their environment) was highly correlated with air temperature (R = 0.97, P < 0.0001), so only temperature data are presented. Ambient daily temperature was obtained from the Australian Bureau of Meteorology for the nearest town to the field site (Olary, SA, 25 km from Bimbowrie).

All active nests we found were recorded (n = 27) including 12 nests during incubation and 15 roost nests (of which 9 roost nests were visited at the time of the recording). Each nest was recorded for up to six recording days, for 2 to 13 h per day (unless the equipment failed after 30 minutes (n = 1) or 1 hour (n = 1)). Presence in the nest and individual sex was determined from calls and noises in the nest recordings, and confirmed with direct observations of the birds. All individuals recorded were fully matured adults (from plumage). A bout was defined as the uninterrupted presence of an individual settled in the nest for more than three minutes (mean duration ± s.e.: 55 ± 4 min). Short nest visits (<3 min, mean duration ± s.e.: 1 ± 0.05 min), typically when an individual repeatedly bring nesting material to its partner in the nest, were not considered as incubation or roosting bouts and excluded from the analyses. Varying that short-visit exclusion criteria from 3 min to either 2 or 5 min did not change any of the results. No individual ever called during these short nest-building visits (n = 172 short visits, in 10 breeding and 7 roosting nests, total 2.9 hours).

### Audio recording in temperature controlled chambers

The chamber, placed in a dark temperature-controlled cabinet, consisted of a 1.5 L clear plastic container fitted with inlet and outlet ports to allow provision of dry air to maintain low humidity levels, similar to those measured in the wild. A tie-clip microphone (as above) was placed inside the chamber, and an infrared video camera (Jaycar, Australia) on the outside. We recorded 20 wild-derived adult zebra finches (10 of each sex), in either morning (starting 10:30am) or afternoon (2:30 pm) sessions, in March 2018. Each bird was recorded twice (except for 1 bird recorded only once because of scheduling constraints), 6 to 24 days apart (average 15 days), starting with either the morning or afternoon session and swapping for the second recording. A bird was caught in its home-cage (at 25 °C), weighted in a cloth bag on a digital scale (to nearest 0.01 g), and placed in the chamber at 25 °C for 25 or 45 min (for first and second recording respectively). Temperature was then increased to 35 °C for 30 min, and then to 40 °C, 42 °C and 44 °C for 20 min at each stage, unless the bird (monitored on the video) showed extreme agitation or weakness and had to be removed from the chamber (i.e. 2 birds were removed at 42 °C).

All procedures in the field and lab were approved by Deakin University Animal Ethics Committee (permit numbers B18-2017 and G06-2017) and the South Australian Department of Environment, Water and Natural Resources (DEWNR; permit U26305-4). All experiments were performed in accordance with Australian guidelines and regulations for the use of animals in research.

### Acoustic analyses

For both field and lab recordings, spectrograms were inspected visually in Adobe Audition (Creative Cloud 2018) for the presence of incubation calls, by an experimenter, blind to the nest/bird identity, date and time of day^12^. The first and second recording per bird in the chamber were analysed by two different experimenters. Start and end time of incubation calling sequences were noted, where calls within 20 seconds of each other were considered as belonging to the same sequence. Sequence durations were summed, and divided by the duration of temperature stage, to obtained the calling rate per minute^12^.

### Statistical analyses

To test predictors for calling occurrence (presence/absence of vocalisations) in the wild and under controlled lab conditions, we used Generalized Linear Mixed Models (*GLMM*s) with a logit link function and a Binomial error distribution (*glmer* function from the *lme4* package in R), and normalised all predictors^36^. For field data (*n* = 177 in-nest bouts from 40 individuals in 21 nests), we used calling occurrence during a bout as the response variable and individual ID as a random effect (estimate for nest ID as a random effect was null). Nest-site temperature during the incubation bout, the linear and quadratic term for the time of day, sex and nest type (incubating or roost) were used as fixed factors. We also tested the interaction between sex and temperature. We could not include both ambient and nest-site temperature in the same model because they were highly correlated, so we included them in alternative models, that we compared using Akaike Information Criteria. For lab data (*n* = 191 temperature stages, for 20 individuals), we used calling occurrence during a temperature stage as the response variable, individual ID as a random effect (estimate for recording day as a random effect was null), and experimental temperature, time of day (morning or afternoon), sex and trial number (first or second per individual) as fixed factors. In addition, we used the same random (including day, non-null) and fixed factors in a GLMM with a logit link function and a Poisson error distribution using calling rate as the response variable. Within individual repeatability in the temperature threshold triggering vocalisation between trials 1 and 2 was tested using a non-parametric Spearman correlation. Lastly, the effect of relative body mass (mass in g over tarsus length in mm), sex and time of day on the calling temperature threshold in trial 1 were tested together using a linear model (*lm* function in R); temperature threshold was raised to the power of 5 to achieve normal distribution of the residuals, as validated by the Shapiro test. All tests were two-tailed. The data is available on Mendeley (10.17632/2krj3kj7k3.1).
